# The mechanism of internal and external efficacy influences residents’ pro-environmental behavior through environmental willingness

**DOI:** 10.1371/journal.pone.0298378

**Published:** 2024-03-01

**Authors:** Qi-Song Yan, Zhao-Qi Zhang, Cai-Xia Er, Wen-Qing Wang

**Affiliations:** 1 School of Law, Politics and Economics, Chongqing University of Science and Technology, Chongqing, *PR* China; 2 School of Sociology, Yunnan Minzu University, Kunming, Yunnan, *PR* China; University of Central Punjab, PAKISTAN

## Abstract

The Chinese government’s environmental conservation efforts require the active participation of all society. This study investigated how internal and external efficacy influence pro-environmental behavior with environmental willingness as a mediator. This study employed a structural equation model to analyze the data from 1499 survey questionnaires. The analysis revealed that both internal and external efficacy can enhance individuals’ pro-environmental behavior in the private and public spheres. External efficacy has a stronger impact on environmental willingness and public sphere environmental behavior, while internal efficacy more significantly influences private sphere environmental behavior. Additionally, environmental willingness only mediates efficacy and public sphere environmental behavior. The innovation of this study is the examination of internal and external efficacy from the perspective of different sources and the comparison of their differential impacts on pro-environmental behavior. Relevant policies should effectively enhance residents’ internal and external efficacy to comprehensively improve their level of pro-environmental behavior.

## 1. Introduction

Over the past four decades since China’s reform and opening up, the country has been striving for modernization, promoting economic growth, and witnessing rapid development in industrialization and urbanization across the nation. However, this progress has also led to environmental challenges due to industrial production, petrochemical fuels, and rapid consumption. To address these issues, the Chinese government has established a framework for building an ecological civilization, which is government-led and involves public participation [1]. Various social entities, including governments at all levels, grassroots community organizations, leaders, and market organizations, have invested substantial resources to collaboratively address environmental pollution. These efforts have strengthened residents’ confidence in pollution governance, and residents were willing to cooperate with external entities such as the government, community organizations, and leaders in environmental governance. At the same time, the Chinese government also encourages the vast majority of residents to engage in environmental protection activities independently. For instance, the government hoped that rural residents would take responsibility for the environmental remediation around their homes and the renovation of toilets. However, the rapid social transformation has led to the disintegration of traditional collective organizations in China and new citizen organizations have not yet been fully established. In this new social context, the behavior of rural residents in managing their living environment largely depends on the individual’s willingness, rather than the norms and constraints of environmental protection by the group or external organization to which the residents belong. For example, the Chinese government requires the implementation of household waste sorting, but the number of residents who actually implement waste sorting is still small [2, 3]. Villagers place more hope on the financial and human resources input from the government and community organizations for toilet quality improvement work, and the enthusiasm of villagers themselves to invest in toilet quality improvement is not high [4, 5]. Why can residents cooperate with the ruling party, government, community organizations, and leaders to carry out environmental governance work in public areas, but the enthusiasm of villagers to independently carry out residential environmental remediation work is not high? The environmental governance led by public institutions needs a lot of resources, which boosts residents’ confidence in the government’s environmental governance. However, the large resource input from public institutions, which is hard for ordinary residents to bear, may affect how they assess their own ability to participate in environmental governance. In other words, does the residents’ perception of the efficacy of environmental governance by public institutions and their own efficacy in environmental governance influence their participation in environmental governance? Is there a difference in how these two perceptions of efficacies influence residents’ environmental behavior? To answer these questions, this article uses the theory of efficacy to compare the different impacts of various types of efficacies on residents’ participation in environmental protection behavior.

Bandura proposed the concept of self-efficacy, which represents an individual’s judgment concerning his or her ability to perform specific tasks [6]. Self-efficacy determines whether individuals will initiate coping behavior, how much effort they will exert, and how long they can maintain this effort when facing obstacles and aversion. As a result, efficacy has become crucial for explaining public willingness and behavior across various research areas [7–10]. Although few studies have shown that people’s self-efficacy does not significantly affect their behavior [11], most studies indicate that self-efficacy positively influences people’s actions [12, 13]. As environmental issues become increasingly prominent, they have gradually become a concern for all countries, and academics are paying attention to public pro-environmental behavior. Some studies have examined the relationship between self-efficacy, public environmental willingness, and pro-environmental behavior. Previous studies have demonstrated that public self-efficacy is directly related to how likely people are to participate in environmental protection and the level of pro-environmental behavior [14–16]. Inspired by Bandura’s concept of self-efficacy, Ajzen proposed the concept of behavioral control in the theory of planned behavior. This concept refers to the actor’s belief in the behavior and is similar to the concept of efficacy [17]. According to this theory, an individual’s behavior is influenced by the available resources and opportunities, and individuals’ perception of being in control of their actions also plays a significant role in determining their behavior [17, 18]. Furthermore, individuals’ perceived difficulty in performing a particular behavior determines their likelihood of engaging in that behavior. The stronger people’s sense of control over their actions is, the more likely they are to engage in these actions. Empirical evidence has shown that the perception of behavioral control correlates positively with pro-environmental behavior [19].

In line with Bandura’s self-efficacy theory, previous research has focused primarily on the association between self-efficacy and pro-environmental behavior. However, as modern society has progressed, solving specific public or global environmental issues that exceed individual capabilities has necessitated the collective efforts of groups or community organizations to address a broader range of environmental challenges [20, 21]. Scholars have noted this phenomenon and introduced concepts such as collective efficacy to explain it. The concept of collective efficacy refers to individuals’ belief in the ability of the group to which they belong or in the collective power to accomplish a particular task [22, 23]. It is believed that people’s perception of collective efficacy plays a crucial role in environmental pollution prevention policies and behaviors [24]. Several pro-environmental behavior studies have drawn on collective efficacy and suggested that the more confident individuals are in managing their community’s environment, the more likely they are to engage in ecological behavior [25]. Compared with self-efficacy, collective efficacy has more substantial predictive power for public ecological behavior [20]. Several studies have argued that collective efficacy only affects ecological behavior after increased self-efficacy [21]. Other scholars have realized that self-efficacy has insufficient explanatory power for individual behavior and have introduced concepts such as external efficacy and government efficacy to explain public political voting behavior and athletes’ competition behavior [10, 26, 27].

The concepts of collective efficacy, governmental efficacy, and external efficacy represent subdivisions of the sense of efficacy from different perspectives. Research on efficacy perception has shifted from evaluating an agent’s internal capacity to assessing the ability of other agents, leading to a subdivision of types of efficacy perception. It is helpful to compare the influence of different types of efficacy on public behavior, which enhances the explanatory power of efficacy for understanding pro-environmental behavior. Regrettably, however, this transition is not entirely thorough because the criteria for categorizing perceptions of efficacy in previous studies do not fully align with the rapidly evolving social reality in China.

There are two interrelated social phenomena in contemporary China. First, the government controls the majority of resources and decision-making power for social affairs, while citizens or social groups have relatively limited access to resources for action and decision-making [28, 29]. Second, Chinese society has entered a stage of rapid population mobility and increasing social mobility of individuals. Traditional community or unit-based cooperative mechanisms have gradually declined while new types of civic organization citizen groups have not yet been fully developed, resulting in less collaboration within social groups and reduced efficacy of collective action [30, 31]. These two phenomena are intertwined, leading to the fact that the government, which has more resources, and community organizations and grassroots cadres have stronger implementation capacity for environmental protection than ordinary social groups. In this situation, the explanatory power of collective efficacy on residents’ pro-environmental behaviors decreases [32, 33]. In addition, although community organizations in Chinese society largely perform duties assigned by the government [34, 35], they are socially autonomous organizations in terms of legal status rather than government organizations. Therefore, residents’ perceptions of the efficacy of pro-environmental behavior by community organizations and their staff cannot be attributed to government efficacy, and the environmental protection capability of community organizations cannot be completely replaced by the government. The inadequacy of previous research can be attributed to insufficient adaptation of the classification criteria for perceptions of efficacy to change Chinese society, which requires a reassessment of these criteria.

With regard to the sources of efficacy, analysis of the sources of residents’ efficacy perceptions show that self-efficacy is predominantly an assessment of actors’ internal capabilities and the effectiveness of their resources, which some studies refer to as internal efficacy [8, 36]. On the other hand, collective efficacy, government efficacy, and residents’ assessment of the behavioral efficacy of community organizations and leaders are are beliefs formed by the actor’s evaluation of resources and capabilities outside of the individual. Therefore, in comparison to an individual actor’s internal efficacy, these various forms of efficacy can be collectively categorized as external efficacy. In this sense, external efficacy encompasses not only government efficacy and collective efficacy but also residents’ efficacy perceptions of community organizations and their staff in environmental protection because these evaluations pertain to the behavioral capacity of external entities. Thus, external efficacy can be regarded as individuals’ belief in the availability of external resources that may enhance performance and play a crucial role in successfully implementing certain behaviors [32, 33]. External resources can include tools, equipment, effective support, favorable working conditions, advantageous starting positions, or other facilitating factors [27], and they are well suited for analyzing public action [8]. Compared to government efficacy and collective efficacy, external efficacy corresponds to an individual actor’s internal efficacy and involves a greater number of source entities, making it more aligned with the actual characteristics of contemporary Chinese society. This is also an innovative aspect of this paper. Compared to the government efficacy and collective efficacy mentioned in previous studies, external efficacy corresponds to an individual actor’s own internal efficacy, and the subjects it contains not only include the government and collective organizations, but also grassroots community organizations, leading cadres, and other subjects in Chinese society. The scope of subjects it covers is different from that in Western societies, and it is more in line with the actual situation of actors in today’s Chinese society. Analysis based on this concept can expand the boundaries of research. Compare with the research done by Western scholars, explain different phenomena, and help carry out cross-cultural comparative research, which is the innovation of this article compared to previous research.

Based on the above discussion, this study divides efficacy into internal efficacy (INE) and external efficacy (EXE). In this study, INE refers to assessing one’s ability to engage in the effects of pro-environmental behavior. In contrast, external efficacy refers to assessing the capacity of social agents other than the self to engage in pro-environmental behavior, including assessing group, community, and local government resources and abilities for environmental protection. Both INE and EXE constitute efficacy and represent the assessment of different entities’ resources, capabilities, and confidence. While the motivating role of efficacy in individual pro-environmental behavior has been explored in previous research in addition to the direct relationship between environmental willingness (EW) and pro-environmental behavior, there has been a lack of comparative analysis of the differences between internal and external efficacy and their relationship with EW. Furthermore, there has been limited examination of the role of EW in the relationship between internal and external efficacy and pro-environmental behavior. Distinctions between people’s PRIEB and PUBEB have been discussed by Stern [37], and there are variations in the effects of different factors on these two types of pro-environmental behavior [38]. However, the distinctions between the two types of pro-environmental behavior in relation to the impact of INE and EXE have not been scrutinized in previous studies.

**Fig 1:**
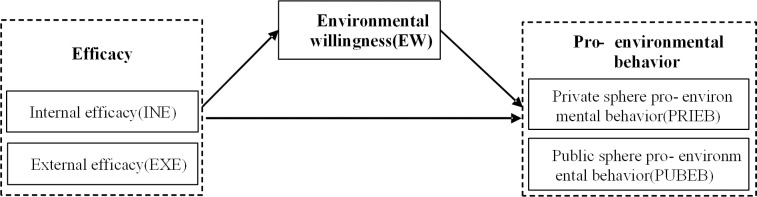
Diagram of the research framework.

This study adopts a perspective that combines INE and EXE to analyze the impact of these factors on both PRIEB and PUBEB. Additionally, the study explores the mediating role of EW in the relationship between INE, EXE, and pro-environmental behavior. Consequently, the analytical framework employed in this study is illustrated in Fig 1.

## 2. Literature review and research hypothesis

### 2.1 Internal and external efficacy and environmental willingness

Environmental willingness (EW) encompasses residents’ inclination to participate in ecological protection behavior, including environmental governance, resource conservation, and purchasing green products. Both the willingness for environmental protection and the willingness to pay for it are encompassed within this concept [39]. Previous research has highlighted the significant role of INE and EXE in shaping individuals’ willingness toward environmental protection, which ultimately influences their actual behaviors. Moreover, these factors can explain up to 30% of the variation observed in individuals’ willingness to pay for environmental protection [40, 41]. The assessment of behavior difficulty, as determined by individuals’ INE and EXE, directly impacts their willingness to take action. When individuals perceive a behavior as less challenging and anticipate favorable outcomes, they are more likely to engage in it. Studies on public green consumption have distinguished between green self-efficacy and consumption efficacy. These studies suggest that green public self-efficacy not only directly influences residents’ willingness to make green purchases but also indirectly affects this willingness through external consumption efficacy [42]. In situations where public environmental problems exert social pressure, the influence of personal INE in determining coping behavior is surpassed by the significance of EXE [20, 40]. When task difficulty falls within a moderate range, rather than being categorized as easy or difficult, participants display heightened levels of external collective efficacy, strengthening their willingness to participate in pro-environmental behavior. Promoting people’s willingness to protect the environment is significantly attributed to external collective efficacy [43]. However, the impact of EXE on EW is subject to certain limitations. In societies characterized by a strong collectivist culture, individuals may experience an intensified sense of environmental obligations imposed by collective culture upon all members of society. This heightened awareness can enhance their EXE in dealing with environmental protection and subsequently reinforce their willingness for environmental preservation [44]. Studies on the governance of environmental degradation also emphasize that while collective efforts are more effective in addressing environmental issues than individual efforts, this effect is restricted to active groups. For nonactive groups, external collective efficacy does not significantly influence EW [45]. These findings are not entirely consistent with conclusions drawn by some previous studies [46]. Overall, public willingness for environmental protection and support for environmental protection policies has been found to be promoted by INE. However, the stability of the relationship between EXE and EW has not been consistently demonstrated and thus warrants further investigation. Based on the above discussion, this study proposes the following hypotheses.

H1a: The higher the level of internal efficacy is, the higher the level of environmental willingness.
H1b: The higher the level of external efficacy is, the higher the level of environmental willingness.
H1c: External efficacy has a more significant promoting effect on environmental willingness than internal efficacy.

### 2.2 Internal and external efficacy and pro-environmental behavior

Bandura posited that individuals assess their ability to perform a specific action before engaging in it [6]. He also argued that people’s motivation, emotional state, and actions are influenced more by their beliefs than by objective circumstances [24]. The greater individuals’ confidence and expectations are in their ability and resources to accomplish a specific behavior, the stronger their self-efficacy. Consequently, this heightened self-efficacy encourages individuals to actively and diligently engage in the behavior [6]. Bandura emphasized that internal cognitive processes, motivation, emotions, and other mechanisms validate individuals’ experiences and ability to control their beliefs [47]. According to Bandura’s reasoning, individuals who are evaluated higher in terms of their internal experiences and resource abilities are likely to have stronger beliefs and expectations regarding environmental protection and improvement. They also exhibit a clearer understanding of the significance and value of protecting and enhancing the environment, resulting in a greater willingness to exert efforts toward environmental conservation. As a result, individuals become more willing to invest effort in environmental protection. Previous empirical studies have demonstrated that positive beliefs stemming from individuals’ assessment of their ability to perform specific behavior contribute to their engagement in pro-environmental behavior [11]. Moreover, the stronger an individual’s positive attitude is toward the role of his or her behavior in improving the ecological environment, the more likely the individual is to undertake corresponding pro-environmental behavior [48]. INE significantly promotes both private and public sphere pro-environmental behavior [21, 49].

On the other hand, EXE primarily pertains to an actor’s evaluation and belief in the ability of social agents other than oneself to accomplish a specific behavior [50]. When individuals perceive that the resources and capabilities of external agents contribute to achieving desired outcomes, they feel supported by these agents, which increases their likelihood of engaging in the behavior. Although these resources and capabilities are external to individuals, they are key reference elements for decision-making. When the public believes that their actions will drive social change or contribute to problem solving, they are more inclined to participate in such behavior [8]. People’s positive shared beliefs in expected outcomes related to ecological and environmental issues, climate change, and other problems can mobilize society’s knowledge, resources, and skills based on collective intentions, facilitating coordinated efforts and mutual assistance to improve environmental quality.

Previous studies have yielded inconsistent findings regarding the relationship between efficacy and pro-environmental behavior. One perspective suggests that residents’ environmental efficacy positively influences their level of pro-environmental behavior. Studies on private sphere resource recycling have found that higher self-assessments of recycling abilities correspond to increased engagement in recycling behavior, and internal efficacy can indirectly impact pro-environmental behavior through other factors [51]. Contrary to the viewpoint above, it has been suggested that a discrepancy exists between individuals’ INE and pro-environmental behavior. In a rapidly evolving society, there are limited social problems that individuals can solve [52], such as public ecological issues and climate change, which necessitate collaborative efforts from interdependent groups, society, and the government to seek solutions and enhance the quality of life [20]. Compared to INE, EXE has been found to be more effective in addressing public environmental problems. When public environmental issues gain prominence in the lives of residents, the meaning, value, and possibility of successful action are perceived through EXE. In other words, it can be argued that EXE has the potential to more consistently promote individuals’ pro-environmental behavior in public environmental issues compared to INE [20, 40]. If the organizations or governments in the community can address local environmental problems, this enhances the public’s confidence in problem solving and increases the likelihood of appropriate pro-environmental behavior [53, 54]. Conversely, individuals’ INE explanatory power appears to be insufficient in addressing public sphere environmental problems. Therefore, this paper proposes the following hypotheses.

H2a: Internal and external efficacy positively promote an individual’s private sphere environmental behavior.
H2b: Internal efficacy has a more positive promoting effect on private sphere environmental behavior than external efficacy.
H2c: Internal and external efficacy positively promote an individual’s public sphere environmental behavior.
H2d: External efficacy has a more positive promoting effect on public sphere environmental behavior than internal efficacy.

### 2.3 INE, EXE, EW, and pro-environmental behavior

Two distinct conclusions have emerged from prior analyses of the relationship between EW and pro-environmental behavior. Most studies support the notion that EW enhances levels of pro-environmental behavior [55]. These studies posit that individuals’ EW serves as the motivating factor that influences their pro-environmental behavior, reflecting their willingness or plans to undertake corresponding actions [17]. Moreover, individual environmental protection efficacy not only positively influences EW but also indirectly facilitates pro-environmental behavior [56, 57]. Previous research has found a positive influence of EW on individuals’ engagement in pro-environmental behavior [58, 59]. When individuals become aware of environmental risks and demonstrate willingness to mitigate them, they are more inclined to engage in pro-environmental behavior provided that the cost of environmental protection remains acceptable [37]. In contrast to the findings above, an alternative viewpoint is proposed by some studies that suggest that pro-environmental behavior is not necessarily entirely aligned with EW. These studies argue that the relationship between EW and pro-environmental behavior is more complex, highlighting a weaker predictive role of EW in relation to pro-environmental behavior [60, 61]. Some studies have even suggested that there is a discrepancy between pro-environmental behavior and EW [62, 63].

This paper argues that although there is a lack of complete consistency between EW and pro-environmental behavior due to situational factors, there is relatively scarce literature to support this viewpoint. Considering the discussions above, it is postulated that both INE and EXE positively influence individuals’ EW, thereby maintaining overall alignment with their pro-environmental behavior. Consequently, EW serves as an intermediary between INE, EXE and pro-environmental behavior. In combination with the content of subhypotheses 1c and 1d presented in Hypothesis 1 of the previous section, the third research hypothesis of this study can be derived.

H3a: Both internal and external efficacy positively affect private sphere environmental behavior through environmental willingness.
H3b: Compared with external efficacy, internal efficacy has a more significant positive indirect effect on private-sphere environmental behavior.
H3c: Both internal and external efficacy positively affect public sphere environmental behavior through environmental willingness.
H3d: External efficacy has a more positive indirect effect on public sphere environmental behavior than internal efficacy.

## 3. Methodology

### 3.1 Sample and data collection

The data used in this paper were obtained from a questionnaire survey conducted in 2020 in the Chinese provinces of Chongqing, Sichuan, Guizhou, Henan, Shanxi, Hubei, Fujian, Shandong, and Guangdong. The study population for this research comprised residents with Chinese citizenship or household registration. The data collection for this study employed a multistage random sampling approach. With the participants’ consent, the surveyors informed them about the survey topic and the confidentiality measures regarding the survey data. Subsequently, the surveyors conducted one-on-one interviews with the participants who completed questionnaires. A total of 1,499 valid questionnaires were collected in this survey. The distribution of variables such as gender, age, education level, annual household income, and place of residence among respondents was diverse, and the sample distribution was representative (Table 1).

**Table 1:** Demographic profile of respondents.

Variable	Value	Frequency	Percentage
Gender	Female	848	56.57
	Male	651	43.43
Age	Under 29 years old	503	33.6
	30–44 years old	415	27.7
	45–59 years old	482	32.2
	Over 60 years old	99	6.6
Educational level	Primary School and Below	178	11.88
	Junior high school	302	20.15
	Senior high school	274	18.28
	Junior college	220	14.68
	Undergraduate	481	32.09
	Postgraduate or Above	44	2.94
Annual household income (RMB)	Below 30,000	248	16.5
	30,000 to 100,000	718	47.9
	100,000 to 200,000	347	23.1
	More than 200,000	186	12.4
Place of residence	Rural	537	35.82
	Urban	962	64.18
Marriage	Unmarried	580	38.69
	Married	919	61.31

### 3.2 Variable measurement

The latent variables in this study include internal efficacy (INE), external efficacy (EXE), environmental willingness (EW), private sphere environmental behavior (PRIEB), and public sphere environmental behavior (PUBEB). This article’s measure of internal efficacy mainly refers to individuals’ capacity assessment of the effectiveness of their own environmental conservation behavior. External efficacy, in contrast, refers to individuals’ capacity assessment of the effectiveness of other social entities apart from themselves in engaging in environmental conservation behavior. The measurement of both internal and external efficacy drew upon methods used by Chamberlain and other scholars [10, 26, 27]. These methods were modified to create a scale specifically tailored to measure efficacy toward environmental conservation behavior. The revised efficacy scale consisted of seven items, such as "I believe that my environmental conservation behavior can contribute to a better environment" and "I believe that local government is quick to address environmental pollution."

In this article, EW refers to residents’ intentions to expend effort for environmental conservation behavior, including environmental intentions and willingness to pay for environmental protection measures such as resource conservation and purchasing green products [39]. The measurement of environmental willingness in this study drew upon the methods used by Kim and Han [64], which consist of three statements, such as "I am not willing to spend time sorting out garbage because it is too tiring."

In this article, private sphere environmental behavior refers to environmental conservation actions conducted within the individual’s personal space, while public sphere environmental behavior refers to environmental conservation actions conducted within public spaces. The measurement of private sphere and public sphere environmental behavior in this study was based on scales borrowed from Kollmuss and other scholars [65, 66] and was modified accordingly. It included seven items, such as "I turn off the lights when I am the last one to leave the room" and "I participate in environmental awareness activities organized by the community." Each item was measured using a 5-point Likert scale, where 1 indicated "completely disagree" and 5 indicated "strongly agree." Higher scores indicated greater agreement of the respondents with the statements.

Table 2 presents the corresponding measurement items for these five latent variables. Each item was measured using a 5-point Likert scale, with 1 indicating "strongly disagree" and 5 indicating "strongly agree." Higher scores indicated greater agreement among the respondents with the statements made. The reliability and validity of these items will be examined and presented in the analysis and results section.

**Table 2:** Latent variables measurement scale.

Latent variable	Items
Internal Efficacy (INE)	I believe that my environmental protection actions can make the environment better (INE1)
	My environmental protection behavior can involve people around me (INE2)
	My environmental protection behavior can be recognized by the government and society (INE3)
External Efficacy (EXE)	The speed of local government to control environmental pollution is fast (EXE1)
	Reasonable and effective measures are taken by local government to control environmental pollution (EXE2)
	Village committees (neighborhood committees) have a good effect in mobilizing the masses to participate in environmental protection (EXE3)
	Cadres take the lead in maintaining the community environment and promoting the participation of community residents (EXE4)
Environmental Willingness (EW)	I do not want to spend time on garbage sorting, and it takes too much energy (EW1)
	I can get involved in environmental protection, but it is better not to spend my money (EW2)
	It is hard for people like me to do anything for the environment (EW3)
Public Sphere Pro-environmental Behavior (PUBEB)	Participate in community-organized environmental awareness activities (PUEB1)
	Maintaining the environmental hygiene of the public areas of the workplace (PUEB2)
	Participating in environmental protection activities organized by NGOs (PUEB3)
	Participating in environmental complaints (PUEB4)
Private Sphere Pro-environmental Behavior (PRIEB)	At home, I often save water (PRIEB1)
	When I am the last one to leave the room, I turn off the lights (PRIEB2)
	I reuse plastic bags (PRIEB3)

### 3.3 Data analysis

This study utilized SPSS 26.0 to conduct frequency analysis for the demographic variables of the sample, and AMOS 26.0 was employed for structural equation modeling (SEM) analysis. SEM is an integration of factor analysis based on path analysis. SEM typically consists of a measurement model and a structural model [67]. The measurement model aims to explain the relationships between latent variables and observed variables. Formula 1 represents the measurement model for endogenous latent variables, while Formula 2 represents the measurement model for exogenous latent variables. The structural model describes the relationships among latent variables (Formula 3). In these three formulas, η and ξ represent endogenous and exogenous latent variables, respectively; λ indicates the influence coefficients of latent variables on observed variables, γ represents the influence of exogenous latent variables on endogenous latent variables, β represents the effects between endogenous latent variables, and ζ represents the residual terms in the structural model.

$$Y=\lambda_{Y}\eta +\varepsilon$$ (1)

$$X=\lambda_{X}\xi +\delta$$ (2)

η=γξ+βη+ζ (3)

Structural equation modeling (SEM) is suitable for research problems with multiple dependent variables and can analyze both direct and indirect effects between multiple variables. In this study, multiple observed indicators were used to measure each latent variable, and the direct and indirect effects between variables were analyzed. Compared to statistical analysis methods such as multiple linear regression and path analysis, SEM is better suited for simultaneously analyzing relationships between multiple variables and examining issues such as measurement model errors [68]. In recent years, SEM has been widely employed in studies related to pro-environmental behavior and environmental governance [69–71], making it advantageous for analyzing the research questions in this paper. In this study, the bootstrap method was employed in SEM to analyze the impact of internal efficacy and external efficacy on pro-environmental behavior in the public and private spheres. The bootstrap method uses a resampling approach to evaluate the robustness of statistical measures and inferential results, which plays an important role in providing reliable parameter estimates, hypothesis testing, and evaluating predictive models in statistical research [72, 73]. In this paper, the number of bootstrap resampling iterations was set to 5000 with a confidence level of 95%.

## 4. Results and analysis

### 4.1 Reliability and validity test

Before testing the hypotheses, it was necessary to test the measurement model’s appropriateness through confirmatory factor analysis (CFA) [68] and obtain the CFA model’s fitness, composite reliability, convergent validity, and discriminant validity results. The CFA results showed that χ^2^/*df* was 2.813, the goodness-of-fit index (GFI) was 0.977, the adjusted goodness-of-fit index (AGFI) was 0.966, the comparative fit index (CFI) was 0.984, and the root mean square error of approximation (RMSEA) was 0.035. These fit statistics were within the recommended standards [74], indicating that the CFA model fit well.

Table 3 presents the statistical results of the CFA. The composite reliability (CR) values ranged from 0.681 to 0.884, all of which were greater than 0.6, indicating that the questionnaire scale had good internal consistency and high CR [75]. The average variance extracted (AVE) evaluated the convergent validity of the latent variables. The standard load values of each observed variable included in each latent variable in Table 3 ranged from 0.627 to 0.851, which met the requirements [76]. The AVE of each latent variable ranged from 0.416 to 0.655, except for the convergent validity of PRIEB, which was less than 0.5. However, if the corresponding latent variable’s CR value exceeds 0.6, it is still within an acceptable range [77, 78]. Therefore, overall, the latent variables in this study had good aggregation for each index and reached an ideal state of convergent validity.

**Table 3:** Convergent validity and composite reliability test of latent variables.

Latent variables	Index	Nonstandard loadings	Standard error	Critical ratio	P	Standard loadings	Standard loadings squared	AVE	CR
Internal efficacy (INE)	INE1	1				0.737	0.543	0.615	0.826
	INE2	1.171	0.041	28.86	***	0.875	0.766		
	INE3	0.87	0.033	26.356	***	0.732	0.536		
External efficacy (EXE)	EXE1	1				0.794	0.630	0.629	0.871
	EXE2	0.998	0.029	34.082	***	0.838	0.702		
	EXE3	1.038	0.031	33.425	***	0.821	0.674		
	EXE4	0.887	0.031	28.393	***	0.714	0.510		
Environmental willingness (EW)	EW1	1				0.805	0.648	0.563	0.794
	EW2	0.953	0.038	25.182	***	0.755	0.570		
	EW3	0.894	0.038	23.772	***	0.686	0.471		
Public sphere environmental behavior (PUBEB)	PUBEB1	1				0.738	0.545	0.654	0.883
	PUBEB2	1.205	0.039	30.931	***	0.869	0.755		
	PUBEB3	1.214	0.043	28.159	***	0.821	0.674		
	PUBEB4	1.148	0.039	29.189	***	0.800	0.640		
Private sphere environmental behavior (PRIEB)	PRIEB1	1				0.638	0.407	0.416	0.681
	PRIEB2	0.757	0.051	14.967	***	0.699	0.489		
	PRIEB3	0.96	0.061	15.788	***	0.593	0.352		

Note: χ^2^/*df* = 2.813, CFI = .977, AGFI = .966, RMSEA = .035, CR = composite reliability; AVE = average variance extracted.

Table 4 shows the results of the discriminant validity test. The values within diagonal brackets represent the square root of AVE, while the nondiagonal values represent the correlation coefficient between the latent variables. When the square root of AVE is greater than the correlation value between the latent variables, it can be concluded that the latent variable had good discriminant validity with other latent variables [75]. The statistical results show that the square root of AVE was greater than the correlations between all latent variables, indicating that each latent variable’s internal consistency in this study was greater than its correlation with other latent variables. This signifies strong discriminant validity among the latent variables.

**Table 4:** Discriminant validity test.

Latent variables	INE	EXE	EW	PUBEB	PRIEB
INE	(0.784)				
EXE	0.603	(0.793)			
EW	0.474	0.422	(0.750)		
PUBEB	0.257	0.301	0.209	(0.809)	
PRIEB	0.161	0.098	0.023	0.264	(0.645)

### 4.2 Common method bias test

During the questionnaire survey stage of this study, one-to-one structured interviews were conducted between the investigator and the respondents to avoid common method bias caused by measurement circumstances. However, since each latent variable was measured using a scale method, Harman’s single-factor analysis method [79] was adopted to ensure the reliability of the data analysis. The results showed that without rotation, the variance explained by the first factor for the item was 34.27%, which was less than 50%, indicating that there was no common method bias in the data of this study.

### 4.3 Structural equation model and hypothesis testing analysis

#### 4.3.1 Model fit

In this study, SEM was proposed to test the hypothesis of the relationship between INE, EXE, EW, PUBEB, and PRIEB. The model was established with PUBEB and PRIEB as dependent variables, and the fit of the SEM was tested. The results showed that *χ*^2^/*df* = 2.616, which was less than the critical value of 3; GFI = 0.979, AGFI = 0.968, CFI = 0.986, all of which were greater than 0.9; and RMSEA = 0.033, which was less than 0.08. The values of each fit index in the model fully met the fitness requirements [74], indicating that the overall fit of the SEM was good.

#### 4.3.2 Hypothesis testing of the influence effect

**Fig 2:**
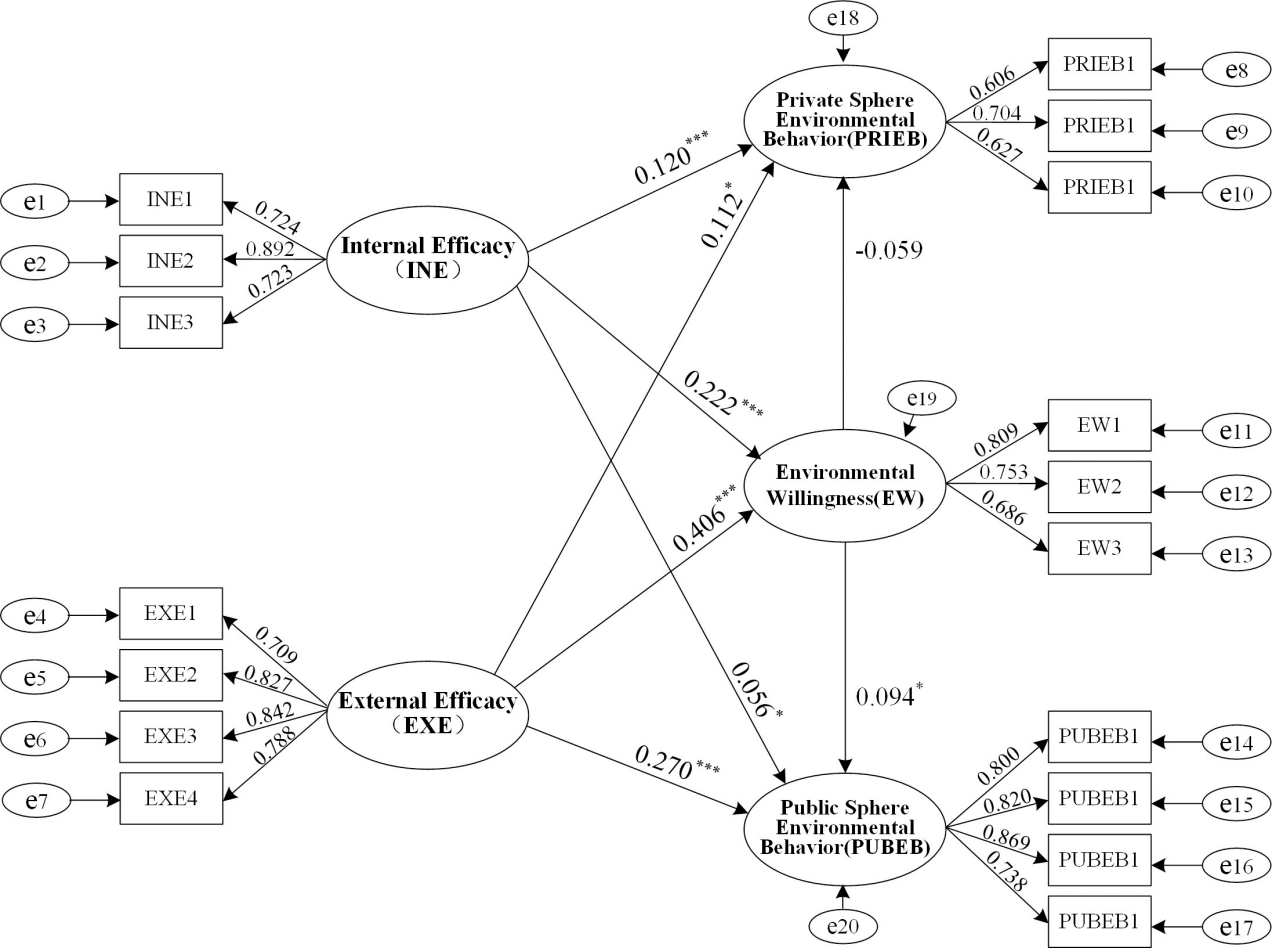
Schematic diagram of SEM results of INE, EXE, EW, and pro-environmental behavior. Note: * p<0.05, ** p<0.01, *** p<0.001.

This study analyzed the effect of INE and EXE on EW, PRIEB, and PUBEB using the bootstrap method in SEM. The number of iterations was set to 5000, and the confidence level was set to 95%. The paths and standardized effect values of INE and EXE on EW, PRIEB, and PEBEB are shown in the path diagram of the SEM presented in Fig 2. Hence, the effect sizes of each path could be directly compared. Fig 2 shows that except for the insignificant effect of EW on PRIEB (β = -0.059, p = 0.211>0.05), all other results in the SEM were significant. Both INE’s effect on EW (β = 0.222, p = 0.001<0.05) and EXE’s (β = 0.406, p = 0.001<0.05) effect on EW had significant positive impacts, verifying Hypotheses 1a and 1b. It was also found that compared with INE, EXE had a greater effect on EW, supporting Hypothesis 1c.

Tables 5 and 6 present the impact effects of internal and external efficacy on PRIEB and PUBEB, respectively. The examination results include the total effects, direct effects, and indirect effects. Analyzing indirect effects helps in understanding the mechanism by which independent variables influence dependent variables. In this paper, analyzing indirect effects enables comparison of the differences in the mechanisms by which INE and EXE influence the two types of pro-environmental behavior, providing a theoretical basis for specific environmental policies. Since the effects were analyzed within the same SEM and Tables 5 and 6 present standardized path coefficients, it is possible to compare the effects of different paths directly.

**Table 5:** Test of direct and indirect effects on PRIEB.

Effect name	Path	β	SE	Lower	Upper	P
Total effect	INE—PRIEB	0.107	0.032	0.046	0.171	0.001
Direct effects	INE—PRIEB	0.120	0.033	0.055	0.185	0.001
Indirect effects	INE—EW—PRIEB	-0.013	0.011	-0.036	0.008	0.197
Total effect	EXE—PRIEB	0.088	0.044	0.001	0.173	0.046
Direct effects	EXE—PRIEB	0.112	0.048	0.013	0.202	0.027
Indirect effects	EXE—EW—PRIEB	-0.024	0.020	-0.064	0.015	0.202

**Table 6:** Test of direct and indirect effects on PUBEB.

Effect name	Path	β	SE	Lower	Upper	P
Total effect	INE—PUBEB	0.076	0.023	0.029	0.120	0.002
Direct effects	INE—PUBEB	0.056	0.023	0.007	0.101	0.020
Indirect effects	INE—EW—PUBEB	0.021	0.008	0.006	0.039	0.008
Total effect	EXE—PUBEB	0.308	0.029	0.252	0.363	0.001
Direct effects	EXE—PUBEB	0.270	0.032	0.209	0.333	0.001
Indirect effects	EXE—EW—PUBEB	0.038	0.015	0.010	0.068	0.011

Table 5 presents the examination results of the total, direct, and indirect effects of internal and external efficacy on PRIEB. According to the data analysis results, both the total effect (β = 0.108, p = 0.001<0.05) and the direct effect (β = 0.122, p = 0.001<0.05) of INE on PRIEB were significant. The total effect (β = 0.089, p = 0.044<0.05) and the direct effect (β = 0.117, p = 0.023<0.05) of EXE on PRIEB were also significant, indicating that both INE and EXE have a positive impact on residents’ PRIEB, verifying Hypothesis 2a. Comparing the total and direct effects of INE and EXE on PRIEB, it was found that the total and direct effects of INE on PRIEB were greater than those of EXE on PRIEB, verifying Hypothesis 2b. The indirect effect of INE on PRIEB was -0.013 (p = 0.197>0.05), while the indirect effect of EXE on PRIEB was -0.024 (p = 0.202>0.05). Both indirect effects were insignificant, so Hypotheses 3a and 3b were not verified. In summary, both INE and EXE positively impact PRIEB through direct influence. Nevertheless, neither has a significant indirect impact on PRIEB through EW as a mediating variable.

Table 6 presents the examination results of the total, direct, and indirect effects of internal and external efficacy on PUBEB. The analysis results revealed that the total effect of internal efficacy on PUBEB (β = 0.076, p = 0.002<0.05) and its direct effect (β = 0.056, p = 0.02<0.05) were significant. The total effect of perceived EXE on PUBEB (β = 0.308, p = 0.001<0.05) and its direct effect (β = 0.270, p = 0.001<0.05) were also significant, verifying Hypothesis 2c. Compared with the direct and total effects of INE on PUBEB, both the total and direct effects of EXE on PUBEB were more significant, verifying Hypothesis 2d. The indirect effect of INE on PUBEB was 0.021 (p = 0.008<0.05), while the indirect effect of EXE on PUBEB was 0.038 (p = 0.011<0.05). Both indirect effects were significant, verifying Hypothesis 3c. The data also showed that compared with INE, EXE had a more effective positive indirect effect on PUBEB, verifying Hypothesis 3d. In summary, both INE and EXE positively affected PUBEB through direct and indirect paths. The results showed that EXE’s total and indirect effects on PUBEB were more significant than those of INE, while the direct effect of INE on PUBEB was more effective than the effect of EXE on PUBEB. This result suggests that compared to EXE, the influence of INE on PUBEB is more reliant on the mediating role of EW.

Based on the above data analysis results, most of the hypotheses in this paper were verified except for H3a and H3b. Table 7 summarizes the specific outcomes of hypothesis validation.

**Table 7:** Results of research hypothesis verification.

Hypothesis	Verified or not
H1a: The higher the level of internal efficacy is, the higher the level of environmental willingness.	Yes
H1b: The higher the level of external efficacy is, the higher the level of environmental willingness.	Yes
H1c: External efficacy has a more significant promoting effect on environmental willingness than internal efficacy.	Yes
H2a: Internal and external efficacy positively promotes an individual’s private sphere environmental behavior.	Yes
H2b: Internal efficacy has a more positive promoting effect on private sphere environmental behavior than external efficacy.	Yes
H2c: Internal and external efficacy positively promotes an individual’s public sphere environmental behavior.	Yes
H2d: External efficacy has a more positive promoting effect on public sphere environmental behavior than internal efficacy.	Yes
H3a: Both internal and external efficacy positively affect private sphere environmental behavior through environmental willingness.	No
H3b: Compared with external efficacy, internal efficacy has a more significant positive indirect effect on private sphere environmental behavior.	No
H3c: Both internal and external efficacy positively affect public sphere environmental behavior through environmental willingness.	Yes
H3d: External efficacy has a more positive indirect effect on public sphere environmental behavior than internal efficacy.	Yes

## 5. Conclusions

### 5.1 Discussion and implications

As part of its vigorous promotion of ecological civilization, Chinese society demands participation and attention from all levels of society. To promote the construction of ecological civilization, individuals are essential components, and whether they are involved in environmental protection depends on their chances of success and their assessment of the impact of their actions. Drawing on Bandura’s self-efficacy theory, this paper examines how efficacy influences pro-environmental behavior through environmental willingness. Compared to previous research, the innovation of this study lies in identifying the inadequacy of the efficacy concept used in prior studies to fully capture the characteristics of contemporary Chinese society and explain residents’ pro-environmental behavior. In response to this limitation, a new concept called "external efficacy" was introduced as a counterpart to internal efficacy. Furthermore, a comparative analysis was conducted to examine the differential impacts of internal and external efficacy on residents’ pro-environmental behavior. This study utilized SEM to assess the total, direct, and indirect effects of INE and EXE on both types of pro-environmental behavior. This research provides new insights by distinguishing between different dimensions of efficacy and pro-environmental behavior. It contributes to understanding the logic behind individuals’ pro-environmental behavior within the context of ecological civilization.

The results of this study indicate that both INE and EXE contribute to pro-environmental behavior in PRIEB and PUBEB. This means that both INE and EXE contribute to PRIEB and PUBEB, disproving the notion that INE does not enhance individuals’ PUBEB [52]. This study demonstrates that both INE and EXE represent the assessment of individuals’ confidence in performing pro-environmental behavior. The distinction lies in the origins of these two types of confidence; the former is derived from an evaluation of one’s own capabilities, while the latter originates from the assessment of the capabilities of entities beyond themselves within society. In this study, it was found that increasing individuals’ INE in environmental protection enables them to implement pro-environmental behavior. This implies that individuals’ environmental literacy should be improved holistically, including increasing actors’ environmental knowledge, raising awareness of environmental protection and environmental risks, and improving skills in solving environmental problems. At the same time, improving individuals’ EXE can also help to improve individual pro-environmental behavior. Environmental protection requires stable support in terms of policies and institutional development, such as government incentives for actors to protect the environment. It also requires the demonstration of external actors in environmental protection actions, such as government, community, leading cadres and other actors taking the lead in all aspects of environmental protection. Additionally, it is important to improve the ability of various groups in Chinese society to collaborate and improve their capability and confidence to solve environmental problems [80].

This paper compares the mechanisms of the effects of INE and EXE on EW and pro-environmental behavior, as opposed to previous studies, which merely examined the effects of INE and EXE on two kinds of pro-environmental behavior. Based on the comparison of EXE with INE, the results indicate that the former significantly affects EW and PUBEB. In contrast, the latter significantly influences only PRIEB. This finding is in line with the initial expectations of this study and corroborates the conclusions of previous related studies [40], further elucidating the differential impact of INE and EXE on EW and various pro-environmental behaviors. This paper explains the reasons for this difference from three perspectives. The first is the psychological support perspective. When actors perceive the ability of external entities, such as the government and community, to protect the environment in public spaces, they consider it an important resource. Actors perceive external entities’ emphasis on environmental protection, which not only supports their behavior regarding environmental protection methods and skills but also motivates them psychologically to participate in environmental preservation. The higher actors’ evaluation of the abilities and beliefs of external entities is regarding environmental protection, the stronger their motivation will be. Although actors perceive this support at a psychological level, it not only regulates their behavioral willingness but also facilitates the transformation of this willingness into actual pro-environmental behavior. The second perspective is that of the resource control structure. The government controls most social resources in China, while individuals have relatively few resources [81, 82]. This resource control structure determines that the construction of an ecological civilization in China is promoted by governments at all levels from top to bottom. However, individuals with few resources can hardly rely on their efforts to promote pro-environmental behavior in the public sphere. In this structural state of resource control, the strength and effectiveness of the government and community in solving environmental problems have substantial reference value for individuals when evaluating their pro-environmental behavior. Individuals have more substantial expectations for the effectiveness of environmental protection by subjects with strong resource control, such as the government and community. Third, contemporary Chinese society exhibits high fluidity from a social mobility perspective. The physical spaces occupied by individuals and their groups are rapidly changing, particularly in urban areas, leading to a high degree of unfamiliarity between individuals. Even in rural Chinese society, villagers rely less on intravillage social capital [83, 84]. While the mobility of contemporary Chinese society has dissolved traditional social support networks among acquaintances, a mature support network based on modern civil society has yet to be established. Furthermore, the level of citizen participation in social governance remains relatively low. As such, individuals have limited confidence in their ability to address public environmental issues and instead look to the government and community organizations to spearhead environmental governance efforts in public spaces.

Given the reasons mentioned above, individuals in contemporary Chinese society are inclined to turn to government, community, and organizational leaders for guidance. When the Chinese government actively promoted the transformation of rural human settlements, cadres in these areas reported a widespread phenomenon of “government path dependence” in environmental governance. This resulted in a situation where "the government is doing, while the villagers are watching." This overreliance on the government is not conducive to mobilizing social members to participate in environmental governance and hinders its sustainable development. In the long run, the stronger the dependence of social individuals on the government is and the greater its investment, the more individuals’ sense of efficacy in participating in environmental protection is eroded. Therefore, it is imperative to effectively coordinate the relationship between INE and EXE to better address environmental issues in the era of globalization.

The results of this study indicate that EW does not necessarily promote all types of pro-environmental behavior. Instead, it only promotes the formation of PUBEB and does not positively affect PRIEB. This study’s conclusions differ from previous research because this study classifies pro-environmental behavior and finds the two conclusions of previous studies in the relationship between two different pro-environmental behaviors and EW [55, 62, 63]. It demonstrates that specific social situations influence the relationship between EW and pro-environmental behavior [85]. In public spaces, individual behavior is subject to the norms of social roles. When actors feel constrained by these norms, their likelihood of transforming EW into PEBEB increases significantly. However, in private spaces, the binding force of social norms on individual behavior is relatively reduced, and individuals do not feel constrained by external social norms on their pro-environmental behavior. In this case, their EW cannot be fully transformed into pro-environmental behavior. If this explanation is valid, it is necessary to strengthen individuals’ perception of social norms to enhance the promoting effect of environmental willingness on private sphere environmental behavior. This can be achieved by internalizing external social norms into individual behavioral norms to facilitate the transformation of personal EW into pro-environmental behavior from within.

### 5.2 Theoretical and policy implications

#### Theoretical implications

Bandura’s self-efficacy theory and Ajzen’s theory of planned behavior emphasize the influence of an individual’s INE on behavior. However, as times have changed, the resolution of environmental problems has extended beyond the scope of individual ability, and greater attention should be given to the impact of EXE on individual pro-environmental behavior. Furthermore, compared to the term “collective efficacy,” EXE is more consistent with the actual social characteristics of contemporary China. It has solid theoretical implications for studying environmental governance in contemporary Chinese society. That is, when examining the pro-environmental behavior of individuals in Chinese society, it is necessary to not only focus on the driving effect of individual psychological characteristics on pro-environmental behavior but also consider China’s historical background. The mobility characteristics of Chinese society, the distribution pattern of political power, and the government’s ability to demonstrate and mobilize society should all be included in analyses of individual pro-environmental behavior. This approach is vital to facilitate a profound examination of individual pro-environmental behavior within the context of Chinese society.

#### Policy implications

This study finds that individuals’ INE is less likely to drive their pro-environmental behavior than EXE. This can lead to dependence on external entities and passive participation in pro-environmental behavior, which is not conducive to implementing the construction of ecological civilization promoted by China among the general public. In China’s process of promoting the construction of ecological civilization, external support for residents’ participation in pro-environmental behavior provided by the government, community, and leadership cadres is undoubtedly important. However, enhancing the possibility of transforming individual internal efficacy into pro-environmental behavior is more important. On the one hand, improving individuals’ environmental literacy, environmental protection skills, and other abilities from the perspective of internal capacity building is necessary to empower individuals with pro-environmental behavior. On the other hand, external support should be given to individuals’ pro-environmental behavior to improve the external guarantee that residents will implement pro-environmental behavior. Only by adopting measures from both aspects can pro-environmental behavior be persistently carried out by residents.

### 5.3 Limitations and suggestions for future research

This study has limitations. First, in line with the characteristics of contemporary Chinese society, this study primarily considers the government, community, and leading cadres as subjects of external efficacy. Due to the limitations of data and other conditions, the collective efficacy of individuals was not included in the analysis of external efficacy in this study. Future studies could compare and analyze the impact of collective and government efficacy on individual pro-environmental behavior. Second, this study is based on Bandura’s self-efficacy theory and Ajzen’s theory of planned behavior. However, it focuses more on the influence mechanism of internal and external efficacy on pro-environmental behavior and does not analyze the impact of individual attitudes and subjective norms on environmental willingness and pro-environmental behavior. Although this approach allows for a more focused examination of the research topic, it is recommended that future research further incorporate all elements of the planned behavior theoretical model, which would help to verify the theoretical model of planned behavior more comprehensively. In future research, the interactive relationship between internal and external efficacy can be explored. Additionally, by comparing research methods, the differential effects of this relationship on environmental protection behavior among residents with different characteristics can be analyzed.

## Supporting information

S1 Data (XLSX)
